# A case study of new assessment and training of unilateral spatial neglect in stroke patients: effect of visual image transformation and visual stimulation by using a head mounted display system (HMD)

**DOI:** 10.1186/1743-0003-7-20

**Published:** 2010-05-16

**Authors:** Toshiaki Tanaka, Tohru Ifukube, Shunichi Sugihara, Takashi Izumi

**Affiliations:** 1 Research Center for Advanced Science and Technology, University of Tokyo, 4-6-1 Komaba, Meguro-ku, Tokyo, 153-8904, Japan; 2 Sapporo Shuyukai Hospital, Shinhassamu 5jo 7chome, Teine-ku, Sapporo, Japan; 3 Department of Human Science and Informatics, School of Biological Science and Engineering, Tokai University, 5-1 Minamisawa, Minami-ku, Sapporo, Japan

## Abstract

**Background:**

Unilateral spatial neglect (USN) is most damaging to an older stroke patient who also has a lower performance in their activities of daily living or those elderly who are still working. The purpose of this study was to understand more accurately pathology of USN using a new HMD system.

**Methods:**

Two stroke patients (Subject A and B) participated in this study after gaining their informed consent and they all had Left USN as determined by clinical tests. Assessments of USN were performed by using the common clinical test (the line cancellation test) and six special tests by using HMD system in the object-centered coordinates (OC) condition and the egocentric coordinates (EC) condition. OC condition focused the test sheet only by a CCD. EC condition was that CCD can always follow the subject's movement. Moreover, the study focused on the effect of the reduced image condition of real image and the arrows.

**Results:**

In Patient A who performed the common test and special tests of OC and EC conditions, the results showed that for the line cancellation test under the common condition, both of the percentage of the correct answers at the right and left sides in the test sheet was 100 percent. However, in the OC condition, the percentage of the correct answers at the left side in the test sheet was 44 percent and the right side was 94 percent. In the EC condition, the left side was 61 percent and the right side was 67 percent. In Patient B, according to the result of the use of reduced image condition and the arrows condition by HMD system, these line cancellation scores more increased than the score of the common test.

**Conclusions:**

The results showed that the assessment of USN using an HMD system may clarify the left neglect area which cannot be easily observed in the clinical evaluation for USN. HMD may be able to produce an artificially versatile environment as compared to the common clinical evaluation and treatment.

## 1. Introduction

Unilateral spatial neglect (USN) is a common syndrome in which a patient fails to report or respond to stimulation from the side of space opposite a brain lesion, where these symptoms are not due to primary sensory or motor deficits [1]. The presence of unilateral spatial neglect has been strongly associated with an increased risk for injury [2] and with poor functional outcome [3]. Clinically, severe unilateral spatial neglect is apparent when a patient collides into his or her surroundings, ignores food on one side of the plate, or attends to only one side of his or her body [4]. Bowen et al. [5] performed a systematic review of published reports. They found 17 reports which directly compared right brain damage (RBD) and left brain damage (LBD) and USN occurs more frequently after RBD than LBD as supported by a systematic review of the published data. However, an accurate estimate of the rates of occurrence and recovery after stroke could not be derived. Several studies have singled out USN as one of the major disruptive factors impeding functional recovery and rehabilitation success [6].

The traditional assessment of USN centers on a variety of simple perceptual motor tasks. Investigations have used line crossing [7], cancellation task [8] and an indented reading test [9]. However, there is no single standardized battery of tests currently available for the assessment of USN.

Previous research has shown that a stationary visual stimulus, such as a letter or digit at the left end of the line, reduces the magnitude of line bisection error [10-12]. Several studies have demonstrated that movement performed in the affected side of space reduces neglect [10,13,14]. A few researchers have suggested that moving visual stimulus may reduce neglect symptom [15,16].

An analysis of USN can be explained with a space coordinate system theory. The boundaries of the neglected space are not constant in as much as the neglect patient's performance is influenced by the relevant system of spatial coordinates; egocentric or object-centered (or allocentric) coordinates. Egocentric coordinate (that is viewer-centered) depends on the object's position relative to the viewer's body, such as trunk, head or eyes. In this frame of reference the terms left and right refer to the observer [17,18]. Object-centered (or allocentric) coordinate on the other hand is a concept that left and right are defined with respect to the object itself [19,20]. Most clinical investigations focused on egocentric (that is viewer-centered) neglect, providing abundant evidence that information is neglected depending on its position relative to body coordinates, e.g. to the retina [21] or trunk [20,22-24]. Patients with unilateral neglect may be influenced by both or either egocentric or allocentric deficiency, but little is known about the neurology and pathology underlying the different frames of reference. Most studies did not discriminate between viewer-centered and object-centered neglect.

Virtual reality (VR) has many advantages over other ADL rehabilitation techniques and offers the potential to enhance a human performance testing and training environment [25]. VR has been investigated in a few studies using devices for augmentation of visual information. For example, there is one approach where head mounted display (HMD) gives a patient with Parkinson' disease an emphasized visual input in order to improve a frozen gait of the patient [26]. HMD has a function which can focus on a certain object or to limit the surrounding environmental conditions, and to offer versatile visual information. Therefore, HMD can produce the object-centred coordinates for a USN patient.

Our previous studies analyzed an evaluation process system of USN in various visual fields using HMD in order to understand more accurately any faults of USN operating in the object-centred coordinates [27,28]. The results showed that the assessment of USN using an HMD system may clarify the left neglected area which cannot be easily observed in the clinical evaluation for USN in the object-centred co-ordinates.

The purpose of this study was to understand more accurately the pathology of USN using a new HMD system in the object-centered co-ordinates and egocentric co-ordinates system. In addition, the study was performed to analyze the effect of transformed visual real image and moving visual stimulation in order to reduce the ignorant area.

## 2. Methods

### 2.1 Patients

Patient A (78 years old) and B (62 years old) who had suffered a stroke (mean age 62 years old) participated in this study after gaining their informed consent. The patients were tested for the presence of any neglect for activities of daily living (ADL) by two physical therapists. Two medical doctors checked the right hemisphere damage of the subject by CT (computed tomography) or MRI (magnetic resonance imaging). Individuals with weak visual acuity, dementia, hemianopsia, apraxia or those being left-handed were excluded from this study. The subject also had to be able to sit on an ordinary chair by him/herself (Table 1).

**Table 1:** Patient characteristics

Subject	Age	Diagnosis	Lesion	Time of rehab. Onset	FIM-M	FIM-C
A: Female	78	1	PT	1 week	42	18
B: Male	62	1	MCA	49 week	58	29

I; infarction. P; parietal lobe, T; temporal lobe

FIM-M; Functional Independence Measure, (Motor Items)

FIM-C; Functional Independence Measure, (Cognitive Items)

MCA: middle cerebral artery territory

*The lesion was right sided

### 2.2 Functional assessment

The Functional Independence Measure (FIM) was executed as an ADL evaluation [29,30]. The FIM motor items scores (FIM-M) and FIM cognitive items scores (FIM-C) were used for the measurement of disability as the best predictors of rehabilitation length of stay for stroke. Two physical therapists evaluated the patients who exhibited specific neglect behaviors in ADL using a special checklist (Table 2).

**Table 2:** Checklist of Everyday Neglect Behaviors

Does the patient:	
1.	Show difficulties when talking or communicating with others?
2.	neglect the left/right side of personal space?
3.	Show difficulties in eating?
4.	Show difficulties in grooming (self-care, washing, bathing, etc.)?
5.	Show difficulties in dressing?
6.	Show difficulties in body movement transferring (from a bed, to W/C, etc.)?
7.	Show difficulties in locomotion 1 (the patient collides against objects and wall on the affected side and/or can not negotiate a W/C between doors, kerbs, etc.)?
8.	Show difficulties in locomotion 2 (the patient turns toward the direction of the affected sid
9.	Show difficulties during PT exercise?
10.	Show difficulties during OT exercise?

A modified version of Halligan's checklist was used [13]. The therapists were requested to score the checklist in terms of those behaviors they considered to be related to visual neglect, as opposed to poor performance that might be expected to follow concomitant disorders such as problems of motor coordination or initiation. The Catherine Bergego Scale (CBS) was also used for assessing neglect behavior [31,32]. The Catherine Bergego Scale (CBS) is based on a direct observation of the patient's functioning in 10 real-life situations, such as grooming, dressing, or wheelchair driving. The CBS includes 10 items that correspond to common everyday life situations. For each item, a 4-point scale was used, ranging from 0 (no neglect) to 3 (severe neglect). A score of 0 was given if no spatial bias was observed; a score of 1 was given in case of a mild neglect, with the patient always exploring right hemispace first, going slowly and hesitatingly toward the left, and showing occasional left-sided omissions; a score of 2 (moderate neglect) was given if the patient showed clear and constant left-sided omissions or collisions; and a score of 3 (severe neglect) was given when a patient was totally unable to explore the left hemispace. A total score was calculated (score range, 0-30). Arbitrary cutoff points were drawn in the total CBS, to distinguish different levels of impairment. The total score indicated mild neglect (score range, 1-10), moderate neglect (score range, 11-20), and severe neglect (score range, 21-30).

## 3. Evaluation for USN

### 3.1 Common clinical test

**Figure 1:**
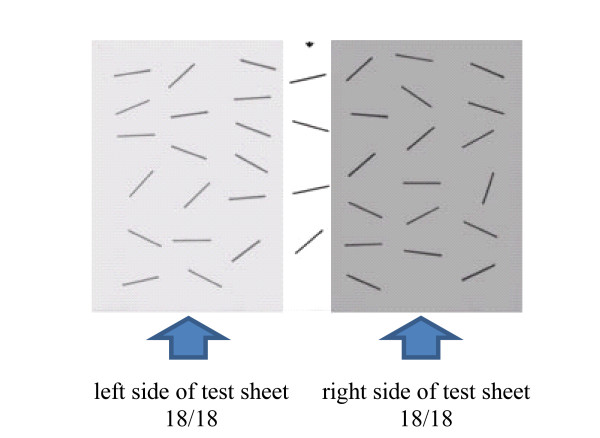
**Analysis method for the line cancellation test**.

To asses neglect, the widely used line and star cancellation tests as included in the Behavioral Inattention Test (BIT) were given to the subjects [33]. We used the line cancellation test of the BIT Japanese version which was modified by Ishiai et al [34]. For the line cancellation test (score range from 0 to 36 points), the subjects were presented with a single sheet of paper on which 6 lines in varying orientations were drawn, 18 on each side. The subjects were instructed to make a mark through all of the lines. Left-sided neglect was indicated by a failure to mark more lines on the left side than on the right. Degree of neglect was assessed by the proportion of lines omitted relative to the total number of lines. The line cancellation test sheet was divided into right and left portions and right and then left correct answer rates were analyzed. A total point score of 34 points was set as a cut-off value (Figure 1).

### 3.2 Special test with Head Mounted Display

#### 3.2.1. Experimental apparatus

The main experimental apparatus includes a CCD Camera (25000 pixel), HMD (FX601, GEOMC), and a digital video camera. HMD's resolution is VGA 640 × 480 (RGB) and it consists of two TFT liquid crystal panels. An image in the display of the HMD was presented to the patient with the CCD camera. Moreover, the patient's head movement was recorded by a digital video camera as means of a qualitative motion analysis.

#### 3.2.2. Assessments of USN with HMD (Figure 2 and 3)

**Figure 2:**
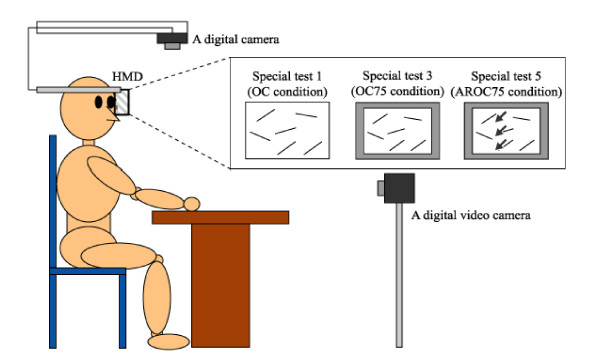
**Experimental setup for special test 1 (OC-ZI condition), 3 (OC-75 condition) and 5 (AROC-75 condition) using the HMD combined system**.

**Figure 3:**
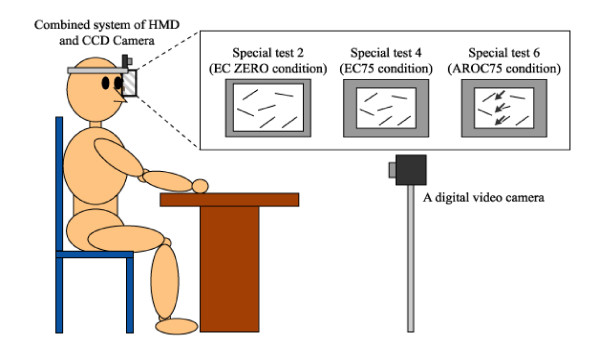
**Experimental setup for special test 2 (EC-ZERO condition), 4 (EC-75 condition) and 6 (AREC-75 condition) using the HMD combined system**.

We attempted to find the grade that USN alters when the coordinate of the patient's visual field was carried out as both object-centered and egocentric coordinates by HMD. Therefore, we used a combined system (CCD camera, HMD, and a computer) in order to display a reduced or an enlarged visual field of real image and then HMD mainly displayed the test sheet to the patient in the form of six special tests that included the following;

a) Special test 1: the zoom-in (ZI) condition which can display only the test sheet using the combined system in the object-centered coordinate (OC-ZI condition).

b) Special test 2: the actual image condition (zero percent of reduction) which the combined system simultaneously moves to follow the patient's own movement in the egocentric coordinate (EC-ZERO condition).

c) Special test 3: the reduced image condition (75 percent of reduction) which can display only the test sheet using the combined system in the object-centered coordinate (OC-75 condition).

d) Special test 4: the reduced image condition (75 percent of reduction) which the combined system simultaneously moves to follow the patient's movement in the egocentric coordinate (EC-75 condition).

Moreover, the arrows that blinked at the left of the display in order to facilitate the patient's attention to the left neglected area were used as a moving visual stimulation and we analyzed the effect of the arrows for improvement of BIT in the condition of special test 3 and 4.

e) Special test 5: the arrows that blinked at the left of the display in order to facilitate the patient's attention to the left neglected area were used in the condition of special test 3 (AROC-75 condition).

f) Special test 6: the arrows that blinked at the left of the display in order to facilitate the patient's attention to the left neglected area were used in the condition of special test 4 (AREC-75 condition).

### 3.3 Procedure

The subjects sat on a wheelchair if needed or on a straight back chair sitting in an up-right position as a starting point. The test sheet was put on a desk and was placed at a midline of each patient's body. All tasks were done without any restriction as to time. The subjects performed the common clinical test and the special test. The subjects were first evaluated by a common clinical test without HMD and then the spatial test with HMD. The line cancellation test was scored using the correct rate and then the score divided into two areas; right and left. The subjects performed the special tests in random order for both object-centered and egocentric co-ordinates. Patient A performed the common clinical test and two special tests; special test 1 and 2 and Patient B performed the common clinical test and four special tests; special test 3, 4, 5 and 6.

## 5. Results

In this study, the score of FIM-M and FIM-C of Patient A and B was 42 and 58, respectively. The scores indicate that the Patients need for maximal or moderate assistance for achieving an adequate performance of ADL.

As the common clinical test for USN, in the first evaluation of the frequency of presence of neglect for ADL for the Patient A and B, USN symptom was existed eight and seven items with ADL, respectively (Table 3). The total CBS score of Patient A and B were 15 and 5 points, respectively. The behavioral neglect of Patient A was categorized moderate and Patient B was categorized as a mild level.

**Table 3:** Ration of USN symptoms in ADL (Patient A and B)

	USN symptoms in ADL	Patient A	Patient B
1.	talking or communicating with others	no existence	no existence
2.	neglecting the left side of bed space	existence	no existence
3.	eating	no existence	no existence
4.	grooming (self-care skills, washing, bathing, etc)	existence	existence
5.	dressing	existence	existence
6.	transferring (from a bed, to W/C, etc)	existence	existence
7.	locomotion1: (negotiating a W/C between doors, kerbs	existence	existence
8.	locomotion2: (the patient turns toward the direction of the affected side.	existence	existence
9.	during PT exercise	existence	existence
10.	during OT exercise	existence	existence

According to the motion analysis of head motion in the common clinical test, Patient A and B began searching from the right side in the line cancellation test. In normal human performance, the head naturally rotated from right to left to follow movement during the line cancellation test. However, the head movement of two patients leftward was insufficient for searching from the right side in all tests.

**Figure 4:**
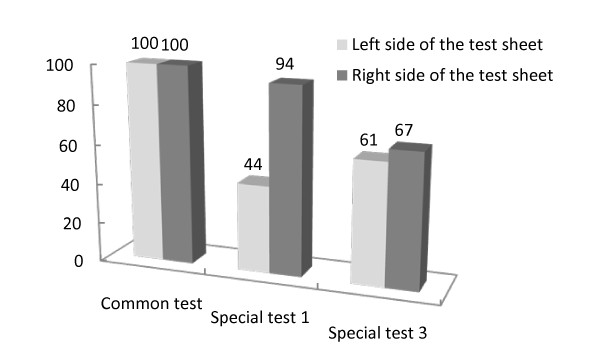
**Percentage of correct answers of the line cancellation test in three tests taken by Patient A; common test, special test 1(OC-ZI condition) and special test 2(EC-ZERO condition)**. Both of the percentage of the correct answers for the right and left sides in the test sheet was 100%. In the special test 1, the percentage of the correct answers at the left side in the test sheet was 44% and the right side was 94%. In the special test 2, the left side was 61% and the right side was 67%.

In Patient A who performed the common test and special test 1, and 2, the results showed that for the line cancellation test under the common condition, both of the percentage of the correct answers for the right and left sides in the test sheet was 100% (Figure 4). However, in the special test 1 (OC-ZI condition), the percentage of the correct answers at the left side in the test sheet was 44% and the right side was 94%. In the special test 2 (EC-ZERO condition), the left side was 61% and the right side was 67% (Figure 4).

**Figure 5:**
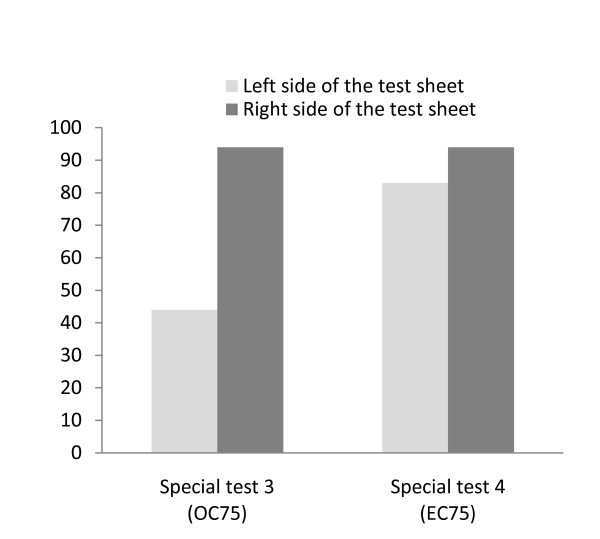
**Percentage of correct answers of the line cancellation test taken by Patient B; special test 3 (OC-75 condition) and 4 (EC-75 condition)**. The percentage of the correct answers at the right side in the test sheet was 100%. The percentage of the correct answers at the left side in the test sheet was 0%. For the line cancellation test under the OC-75 condition in special test 3 with HMD, the correct answer at the left side in the test sheet was 44%. For the EC-75 condition in special test 4 with HMD, the correct answer at the left side was 83%.

**Figure 6:**
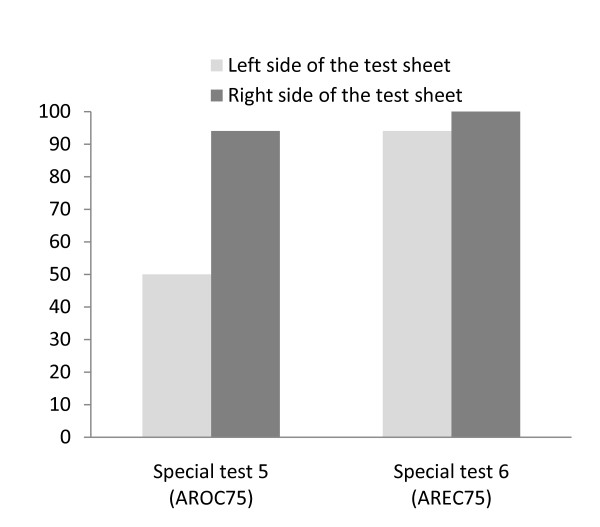
**Percentage of correct answers of the line cancellation test taken by Patient B; special test 5 (AROC-75 condition)and 6 (AREC-75 condition)**. According to the results when the uses of the arrows were employed, in special test 5, the percentage of the correct answers at the left side in the test sheet was 50%. In special test 6, the left side was 94%.

In Patient B, the results showed that for the line cancellation test under the common condition, the percentage of the correct answers at the right side in the test sheet was 100%. However, the percentage of the correct answers at the left side in the test sheet was 0%. For the line cancellation test under the OC-75 condition 75% of reduced real image and OC condition) in special test 3 with HMD, the correct answer at the left side in the test sheet was 44% while the right side was 94% (Figure 5). For the EC-75 condition (75% of reduced real image and EC condition) in special test 4 with HMD, the correct answer at the left side in the test sheet was 83% while the right side was 94% (Figure 5). According to the results when the uses of the arrows were employed, in special test 5 (75% of reduced real image and OC condition: AROC-75 condition), the percentage of the correct answer at the left side in the test sheet was 50% and the right side was 94% (Figure 6). In special test 6 (75% of reduced real image and EC condition: AREC-75 condition), the left side was 94% and the right side was 100% (Figure 6).

## 5. Discussion

For the results of Patient A, the cancellation test of common test was 100% score. However, USN symptom was existed eight items in activities for the frequency of presence of neglect for ADL. The area of neglect had a profound effect on dynamic ADL, for example, dressing, transferring, and locomotion. The common cancellation test did not indicate problems of ADL in relation to the patient's neglect. The subjects' dressing, transferring, and locomotion of checklist by Halligan et al. [13] indicated a high frequency of presence of USN symptoms. The line cancellation score of special test 1 and 2 was lower than that of the common test. When the patients with USN concentrated on an object in OC condition, their USN symptoms were more aggravated for the left test sheet as compared to the right test sheet. For the EC condition, both right and left test sheet score in special test 2 were lower than that in the common test. Patient A had a bias to the right space, because the movement of HMD and CCD camera was synchronized with the subject's head movement. Moreover, the Patient moved her head to find the sheet, and then she might have lost sight of both right and left sheets on the display of HMD. The HMD test may be better able to find a USN symptom which may not be easily detected. This means that the new HMD system might be accurate assess the Patient's problem of USN. The common test score does not match to the occurrence of USN in her ADLs. By evaluating the score in both object-centered coordinates and egocentric coordinates system, the HMD may be able to clarify that each patient has a versatile problem of USN in relation to ADL.

The results also showed that HMD evaluation could produce the condition of an object-centred coordinate and egocentric coordinate system to further clarify the left neglect area which cannot be easily observed in the clinical evaluation for USN. The results of Patient B showed that for the line cancellation test under the common condition, the percentage of the correct answers at the right side and left sides in the test sheet were 100% and 0%, respectively. USN symptom could be found in seven items as activities for the frequency of presence of neglect for ADL. The neglected area increased in size when the subject uses dynamic movement for ADLs, for example, dressing, transferring, and locomotion.

In special test 3 and 4, both scores increased to a level more than the score of the common test. In our former study, the use of the HMD improved the neglect symptoms in all subjects who had right cerebral hemisphere damage [35]. Our HMD system was able to produce a reduced picture of a real image. The patients with USN were assessed by the cancellation test under 4 conditions: 70%, 80%, 90% of reduced visual display and the real image (no reduced visual display) by the HMD. The results showed that the cancellation score of 90% and 80% conditions were significantly greater than that of the real image condition. In this study, we used 75% of reduced image condition because Patient A and B obtained the maximal score of the line cancellation test by 75% condition.

Rossetti et al. [36] investigated the effect of prism adaptation on neglect symptoms, including the pathological shift from the subjective midline to the right. They reported that all patients exposed to the optical shift of the visual field to the right improved in their manual body-midline demonstration and on their classical neuropsychological tests. Lee [37], Woo and Mandelmant [38] also suggested the effectiveness of the Fresnel prism when placed on a spectacle lens for improving various visual-field losses. The improvement induced by the HMD indicates that a signal is given to the brain that stimulates the natural recovery process in the same manner as the prism adaptation method. Moreover, the HMD system may lead to the further correction of left side neglect than a Fresnel prism placed on a spectacle lens. Since a high power Fresnel prism membrane for obtaining a wide field of view is not clear, the prism produces a distortion of a real image and has lowered capabilities of visual acuity. By contrast, the HMD has the possibility of obtaining various fields of view without any deterioration of visual acuity.

According to the results of special test 5 and 6 for Patient B with the dynamic visual stimulation by arrows, both scores increased more than the score of the common test. Moreover, special test 5 and 6 scores were greater than those of special test 3 and 4 at the left side of the test sheet. For the results of the percentage of the correct answers with the use of arrows in the EC condition, both correct answers of the right and left sides were greater than those of the right and left sides without arrows. However, in the OC condition, both correct answers of the right and left sides were almost the same as those of the right and left sides without arrows. This means that the arrows might be helpful to pay more attention to the ignorant area if the display on HMD can allow for movement of the subject's head and trunk like that of an EC condition. In the OC condition, even if using an arrow mode, the subject may not be able to find the ignorant area because the subject's movement cannot match the fixed displays which only focus on the test sheet. In near future, we need to analyze the movements of eyes, head and trunk simultaneously during a common test and the two special tests with or without the arrow's mode to make sure that a patient with USN can pay attention to the ignorant area by the arrow's mode.

Other studies have demonstrated the effect of stationary or moving visual stimulus reduces neglect. Plummer [16] investigated whether the spatial charac-teristics or general alerting properties of moving visual stimuli are responsible for reducing neglect. The results provided evidence that spatial characteristics rather than general alerting properties of moving visual stimuli reduce rightward bisection errors in unilateral neglect. Clinically, it could be argued that visual cues that rely upon instructions to direct the patient's attention toward the neglected side are of limited practical value because they are dependent upon a therapist. In contrast, moving visual stimuli may capture attention automatically, thereby eliminating the need for a therapist to guide the performance. Left-sided and leftward moving visual stimuli can assist in directing attention to the left [15,39]. These effects occur automatically, without any input from another person. Moreover, Butter and Kirsch [40] used moving visual stimuli to investigate whether lateralised moving visual stimuli would enhance neglect patient's search performance in a cancellation task presented on a computer screen. They found that when moving visual cues were presented on the left part of the screen, neglect patients had improved detection of targets on the left.

The HMD system may play an important role in the neuropsychological rehabilitation of unilateral spatial neglect as an evaluation device. Bowen et al. [5] suggested that different USN disorders may exist, which may require type-specific rehabilitation approaches. Our system may have clinical implication for a new assessment because HMD can change versatile visual input to fit each patient's degree of USN. In addition, clinical assessment methods for USN may be able to use various images in HMD by a computer such as change of colors and partial enlargement or reduction of real image, and to produce suitable visual stimulation in HMD for each patient who has USN. In near future, we will try to analyze the use of HMD to assess serially the improvement of patients with USN.

In conclusion, the results showed that the assessment of USN using an HMD system may clarify the left neglect area which cannot be easily observed in the clinical evaluation for USN. Moreover, it might be hypothesized that the USN test using HMD may display a greater accuracy and be able to assess the occurrence and grade of USN to a greater degree more than the common clinical test. HMD can produce an artificially versatile environment as compared to the common clinical evaluation and treatment.

## Competing interests

The authors declare that they have no competing interests.

## Authors' contributions

TT performed the design of this study, acquisition and analysis of data and drafting the manuscript. SS made substantial contribution to acquisition and analysis of the data. T. Ifukube and T. Izumi were involved in conception and design of the study, interpretation of the data and revision of the manuscript for important intellectual content. Each of the authors has read and concurs with the content in the final manuscript. Nobody who qualifies for authorship has been omitted from the list.

## References

[B1] Heilman KM Heilman KM, Valenstein E Neglect and related disorders Clinical Neuropsychology 1979 New York: Oxford University Press

[B2] Ugur C Gucuyener D Uzuner N Ozkan S Ozdemir G Characteristics of falling in patients with stroke J Neurol Neurosurg Psychiatry 2000 69 649 651 10.1136/jnnp.69.5.649 11032620 PMC1763385

[B3] Jehkonen M Ahonen JP Dastidar P Visual neglect as a predictor of functional outcome one year after stroke Acta Neurol Scand 2000 101 195 201 10.1034/j.1600-0404.2000.101003195.x 10705943

[B4] Menon-Nair A Korner-Bitensky N Wood-Dauphinee S Assessment of unilateral spatial neglect post stroke in Canadian acute care hospitals: are we neglecting neglect? Clinical Rehabilitaion 2006 20 623 634 10.1191/0269215506cr974oa 16894806

[B5] Bowen A McKenma K Tallis C Reasons for variability in the reported rate of occurrence of unilateral spatial neglect after stroke Stroke 1999 30 1196 1202 10356099 10.1161/01.str.30.6.1196

[B6] Denes G Semenza C Stoppa E Lis A Unilateral spatial neglect and recovery from hemiplegia: follow-up study Brain 1982 105 543 552 10.1093/brain/105.3.543 7104665

[B7] Albert ML Simple test of visual neglect Neurology 1973 23 658 664 4736313 10.1212/wnl.23.6.658

[B8] Diller L Weinberg J Hemi-inattention in rehabilitation: evolution of rational remediation program Adv Neurol 1977 18 63 82 920526

[B9] Calpan B Assessment of unilateral neglect: a new reading test J Clinical and Experimental Neuropsychology 1987 9 359 364 10.1080/01688638708405056 3597728

[B10] Lin K-C Cermak SA Kinsbourne M Trombly CA Effects of left-sided movements on line bisection in unilateral neglect Journal of the International Neuropsychological Society 1996 2 404 411 10.1017/S135561770000148X 9375165

[B11] Nichelli P Rinaldi M Cubelli R Selective spatial attention and length representation in normal subjects and in patients with unilateral spatial neglect Brain and Cognition 1989 9 57 70 10.1016/0278-2626(89)90044-4 2912475

[B12] Reuter-Lorenz PA Posner MI Components of neglect from right-hemisphere damage: An analysis of line bisection Neuropsychologia 1990 28 4 327 333 10.1016/0028-3932(90)90059-W 2342639

[B13] Halligan PW Cockburn J Wilson BA The behavioural assessment of visual neglect Neuropsychological Rehabilitation 1991 1 1 5 32 10.1080/09602019108401377

[B14] Halligan PW Manning L Marshall JC Hemispheric activation vs spatio-motor cueing in visual neglect: A case study Neuropsychologia 1991 29 2 165 176 10.1016/0028-3932(91)90018-4 2027432

[B15] Butter CM Kirsch NL Reeves G The effect of lateralized dynamic stimuli on unilateral spatial neglect following right hemisphere lesions Restorative Neurology and Neuroscience 1990 2 39 46 10.3233/RNN-1990-2105 21551871

[B16] Plummer P Dunai J Morris ME Understanding the effects of moving visual stimuli on unilateral neglect following stroke Brain and Cognition 2006 60 156 165 10.1016/j.bandc.2005.11.001 16466838

[B17] Karnath HO Disturbed coordinate transformation in the neural representation of space as the crucial mechanism leading to neglect Neuropsychological Rehabilitation 1994 4 147 150 10.1080/09602019408402273

[B18] Karnath HO Subjective body orientation in neglect and the interactive contribution of neck mauscle proprioception and vestibular stimulation Brain 1994 117 1001 1012 10.1093/brain/117.5.1001 7953584

[B19] Marr D Vision 1982 H. Freemann and Co

[B20] Mennemeier M Chatterjee A Heilman KM A comparison of the influences of body and environment centred reference frames on neglect Brain 1994 117 Pt 5 1013 1021 10.1093/brain/117.5.1013 7953585

[B21] Hillis AE Rapp B Benzing L Caramazza A Dissociable coordinate frames of unilateral spatial neglect: "Viewer-centered" neglect Brain and Cognition 1998 37 491 526 10.1006/brcg.1998.1010 9733562

[B22] Beschin N Cubelli R Della Sala S Spinazzola L Left of what? The role of egocentric coordinates in neglect Journal of Neurology, Neurosurgery, and Psychiatry 1997 63 483 489 10.1136/jnnp.63.4.483 9343128 PMC2169750

[B23] Chokron S Right parietal lesions, unilateral spatial neglect, and the egocentric frame of reference NeuroImage 2003 20 75 81 10.1016/j.neuroimage.2003.09.002 14597299

[B24] Karnath HO Spatial orientation and the representation of space with parietal lobe lesions Philosophical Transactions of the Royal Society of London Series B, Biological Sciences 1997 352 1411 1419 10.1098/rstb.1997.0127 9368929 PMC1692059

[B25] Lee JH Ku J Cho W A virtual reality system for the assessment and rehabilitation of the activities of daily living Cyberpsychol Behav 2003 6 4 383 388 10.1089/109493103322278763 14511450

[B26] Prothero JD The treatment of akinesia using virtual images Master's the-sis 1993 Industrial Engineering, University of Washington

[B27] Tanaka T Nara H Ino S Ifukube T Clinical Application of Head Mounted Display System for Left Unilateral Spatial Neglect The Japanese Journal of Ergonomics 2005 41 4 213 217

[B28] Tanaka Toshiaki Sugihara Shunichi Nara Hiroyuki Ino Shuichi Ifukube Tohru A preliminary study of clinical assessment of left unilateral spatial neglect using a head mounted display system (HMD) in rehabilitation engineering technology Journal of NeuroEngineering and Rehabilitation 2005 2 31 10.1186/1743-0003-2-31 16207373 PMC1277843

[B29] Granger CV Hamilton BB Keith RA Zielezny M Sherwin FS Advances in functional assessment for medical rehabilitation Top Geriartr Rehabil 1986 1 3 59 74

[B30] Granger CV Hamilton BB Sherwin FS Eisenberg MG, Grzesiak RC The functional independence measure: a new tool for rehabilitation Advances in Clinical Rehabilitation 1987 New York: Springer-Verlag 3503663

[B31] Azouvi P Marchal F Samuel C Functional consequences and awareness of unilateral neglect: study of an evaluation scale Neuropsychol Rehabil 1996 6 133 50 10.1080/713755501

[B32] Bergego C Azouvi P Samuel C Validation d'une échelle d'évaluation fonctionnelle de l'héminégligence dans la vie quotidienne: l'échelle CB Ann Réadaptation Med Phys 1995 38 183 189 10.1016/0168-6054(96)89317-2

[B33] Wilson BA Cockburn J Halligan PW Behavioural inattention test 1987 Thames Valley Test Company, England

[B34] Ishiai S Behavioural inattention test Japanese ed 1999 Shinkoh Igaku Shuppan, Co., Ltd, Tokyo

[B35] Tanaka T Shirogane S Ohyanagi T Izumi T Yumoto H Ino S Ifukube T Application of head mounted display system for left unilateral special neglect14th International Congress of the World Confederation for Physical Therapy, Proceedings 2003 RR-PO-0982

[B36] Rossetti Y Rode G Pisella L Farne A Li L Boisson D Perenin M-T Prism adaptation to a rightward optical deviation rehabilitates left hemispatial neglect Nature 1998 395 166 169 10.1038/25988 9744273

[B37] Lee AG Perez AM Improving awareness of peripheral visual field using sectorial prism J Am Optom Assoc 1999 70 624 628 10561920

[B38] Woo GC Mandelman T Fresnel prism therapy for right hemianopia Am J Optom & Physiol Optics 1983 60 8 739 743 10.1097/00006324-198308000-00012 6624873

[B39] Mattingley JB Bradshaw JL Bradshaw JA Horizontal visual motion modulates focal attention in left unilateral spatial neglect Journal of Neurology, Neurosurgery, and Psychiatry 1994 57 1228 1235 10.1136/jnnp.57.10.1228 7931385 PMC485492

[B40] Butter CM Kirsch NL Effect of lateralised kinetic visual cues on visual search in patients with unilateral spatial neglect Journal of Clinical and Experimental Neuropsychology 1995 17 6 856 867 10.1080/01688639508402435 8847392

